# Global surgery in a postconflict setting - 5-year results of implementation in the Russian North Caucasus

**DOI:** 10.3402/gha.v8.29227

**Published:** 2015-10-23

**Authors:** Fatima I. Lunze, Karsten Lunze, Zemfira M. Tsorieva, Constantin T. Esenov, Alexandr Reutov, Thomas Eichhorn, Christian Offergeld

**Affiliations:** 1 Department of Cardiology, Boston Children's Hospital, Harvard Medical School, Boston, MA, USA; 2 Health for the Caucasus e.V., Cottbus, Germany; 3 Department of Medicine, Boston University School of Medicine, Boston, MA, USA; 4 Department of Surgery, Vladikavkaz Children's Hospital, Vladikavkaz, Russian Federation; 5 Ministry of Health North Ossetia, Vladikavkaz, Russian Federation; 6 Federal Hospital Beslan, Beslan, Russian Federation; 7 ENT Department, Carl Thiem Hospital Cottbus, Cottbus, Germany; 8 ENT Department, Freiburg University, Freiburg im Breisgau, Germany

**Keywords:** humanitarian surgery, capacity building, mid-income countries, terrorism, ear–nose–throat surgery, otologic surgery, otologic trauma

## Abstract

**Background:**

Collaborations for global surgery face many challenges to achieve fair and safe patient care and to build sustainable capacity. The 2004 terrorist attack on a school in Beslan in North Ossetia in the Russian North Caucasus left many victims with complex otologic barotrauma. In response, we implemented a global surgery partnership between the Vladikavkaz Children's Hospital, international surgical teams, the North Ossetian Health Ministry, and civil society organizations. This study's aim was to describe the implementation and 5-year results of capacity building for complex surgery in a postconflict, mid-income setting.

**Design:**

We conducted an observational study at the Children's Hospital in Vladikavkaz in the autonomous Republic of North Ossetia-Alania, part of the Russian Federation. We assessed the outcomes of 15 initial patients who received otologic surgeries for complex barotrauma resulting from the Beslan terrorism attack and for other indications, and report the incidence of intra- and postoperative complications.

**Results:**

Patients were treated for trauma related to terrorism (53%) and for indications not related to violence (47%). None of the patients developed peri- or postoperative complications. Three patients (two victims of terrorism) who underwent repair of tympanic perforations presented with re-perforations. Four junior and senior surgeons were trained on-site and in Germany to perform and teach similar procedures autonomously.

**Conclusions:**

In mid-income, postconflict settings, complex surgery can be safely implemented and achieve patient outcomes comparable to global standards. Capacity building can build on existing resources, such as operation room management, nursing, and anesthesia services. In postconflict environments, substantial surgical burden is not directly attributable to conflict-related injury and disease, but to health systems weakened by conflicts. Extending training and safe surgical care to include specialized interventions such as microsurgery are integral components to strengthen local capacity and ownership. Our experience identified strategies for fair patient selection and might provide a model for potentially sustainable surgical system building in postconflict environments.

**Table:** 

Фæндараст!.. Фервæзтæ нæ мæтæй – Хуыцауы мацæмæй уал дом! –
Мæрдты дын хай уыдзæн дзæнæтæй,
Уæлæуыл баззайдзæн дæ ном.
	Kosta Khetagurov, Ossetian Poet, 1891

In 2004, a terrorist attack on a school in Beslan in the Republic of North Ossetia-Alania (North Ossetia), an autonomous republic in the North Caucasus region at the south border of the Russian Federation (Russia), killed 334 people and left many more with otologic blast injuries caused by indoor bomb explosions. In response to an appeal from local surgeons to serve these victims, we conducted a needs assessment (1) and initiated a partnership between the Vladikavkaz Children's Hospital and German medical providers, which was facilitated and supported by the Ministry of Health of North Ossetia-Alania and local civil society organizations. Following the suggestion of surgeons in Vladikavkaz who lacked the capacity to provide middle-ear surgery, we focused initial efforts on otologic operations and training of providers in microsurgery techniques, since many victims presented with sequelae of untreated complex ear injuries requiring specialized microsurgery.

No microsurgery services existed in the Russian North Caucasus to address the need of Beslan victims or other patients for treatment. We therefore decided to initiate a global surgery partnership in Vladikavkaz, the capital city of North Ossetia, consistent with a recent definition of global surgery as ‘art and science of surgical practice in pursuit of excellent patient care through mitigation of the inequity in the distribution of worldwide surgical resources’ (2). This includes mid-income settings in post-Soviet countries such as the North Caucasus, where basic surgical and anesthesiology services exist. However, public healthcare services in these settings often lack up-to-date equipment and adequately trained providers.

In contrast to its abundant natural and cultural wealth, the North Caucasus remains an economically disadvantaged region confronted with human rights violations and violence from armed opposition groups (3). Since the end of the Chechen wars in 2000 and the official end of the ensuing insurgency and counterterrorism phase 5 years ago, violence has spread from Chechnya to adjacent republics in the North Caucasus such as North Ossetia and others (4). The 2008 war between Russia and Georgia over South Ossetia, attacks from militant groups in North Ossetia, Ingushetia, Kabardino-Balkaria, and Dagestan, to name but a few examples, illustrate the political instability of the region. Most international organizations and non-governmental organizations have withdrawn from the North Caucasus region due to security concerns. In the last year alone, more than 500 people died from violence between militants and security forces in the North Caucasus (5). The death toll among the 127 Russian security officers killed is on a scale of the 160 soldiers who died during the same period in NATO's war in Afghanistan (6).

The Beslan school hostage crisis by a group of armed Islamic separatist militants had received an appropriate acute trauma response through the local health system's existing general surgical services (1). However, due to the lack of specialized surgery in the region, barotraumatic middle-ear lesions resulting from bomb explosions remained untreated in numerous surviving victims. Otologic lesions are the most common explosion-related injuries (7). Tympanic lesions left untreated can cause different pathological reactions in the mucosal and bony structures of the middle ear, creating an indication for extensive microsurgical reconstruction procedures. In many cases, the lack of treatment for Beslan victims led to tympanosclerotic changes in the middle ear, chronic otitis media, or even cholesteatoma. This requires surgical eradication of inflammatory disease and eventually extensive microsurgical reconstruction of the tympanic membrane, including insertion of partial or total middle-ear prostheses for ossiculoplasty to achieve restoration of middle-ear transfer function. The expertise necessary for these microsurgical procedures is typically found only at major medical centers in developed settings and was absent in the North Caucasus.

Health systems in the North Caucasus, operating in one of the economically most disadvantaged environments in the Russian Federation, are weakened from past conflicts. The wars and subsequent violence hampered medical education and investments in healthcare infrastructure, and without adequate response to address current insufficiencies. According to an OECD review of the Russian health system, North Ossetia ranks lowest of all regions in the Russian Federation with little over Ruble 3,400 in annual public healthcare spending per capita (less than US$ 70 at the time of study, and less than half of what the government in postwar Chechnya spends on health) (8). This budget does not address the need for domestic infrastructure and education of specialists to provide the complex ENT (ear, nose, throat) surgery needed for the victims of terrorism. We therefore initially arranged for individual victims to be treated abroad, and this was expensive. Since treatment abroad did not meet existing needs and was not sustainable, North Ossetian surgeons (ZMT, CTE, and AR) advocated for local capacity building and sought authorization from the regional health ministry to cooperate with an international humanitarian surgical team, who in turn obtained approval from the Russian Federal Government to establish operations in Vladikavkaz. The goal of this study is to describe our program implementation strategies, experiences, and patient outcomes after 5 years of capacity building.

## Methods

### Procedures, patient selection, and data collection

To ensure local ownership of the collaboration and appropriate procedures in consideration of existing patient care services and teaching activities, North Ossetian surgeons led patient selection and follow-up. Preceding the initial operations described here, two North Ossetian surgeons (including ZMT) received theoretical and practical, hands-on operating room (OR) training in otologic microsurgical procedures in Germany (Carl-Thiem Hospital, Cottbus, under the supervision of TE, head of the department of ENT). These surgeons were trained based on their prior medical education in a 6-year medical school curriculum and a 3-year or longer surgical training (1 year internatur and 2 years ordinatur) at the North Ossetian Medical Faculty (SOGMA) or other fully accredited institutions of higher medical education in the Russian Federation. No specialized training in ENT surgery or other dedicated subspecialty training is available in Vladikavkaz. Following their general surgical education, practitioners usually train at major centers elsewhere in Russia or abroad for a longer period of time, to the extent that they can independently lead an outpatient clinic with minor ENT diagnostics and interventions.

Specialized training provided through the project enabled local surgeons to identify suitable patients, assist with operations on-site, perform minor specialized surgery, and provide postoperative care in consultation with a remotely available specialist. The local surgeons also directed the coordination of all steps in North Ossetia, including the collaboration of existing pediatric and adult surgical, nursing, and anesthesiology services.

In preparation of the project, supported by the German NGOs ‘Kindernothilfe’ and ‘Deutsche Diakonie’, we purchased a dedicated operation microscope and necessary specialized operation equipment and consumables from manufacturers in Germany and transported them to the Children's Hospital Vladikavkaz. We also founded the Germany-based, non-profit civil society organization ‘Health for the Caucasus’ with the goal to support scale-up, funding and management of our partnership activities in the region, including countries in the North and South Caucasus. During subsequent activities in North Ossetia, consumables were provided by the Children's Hospital Vladikavkaz and other collaborating facilities.

We evaluated patients presenting to Vladikavkaz Children's Hospital, provided preoperative assessment and care, obtained informed consent where operations were indicated, and accordingly planned for OR capacities. To ensure a fair and equitable patient selection, we consulted civil society groups such as the Beslan Citizen Committee and other victims’ representatives’ organizations to identify Beslan victims and other patients in need.

**Fig. 1:**
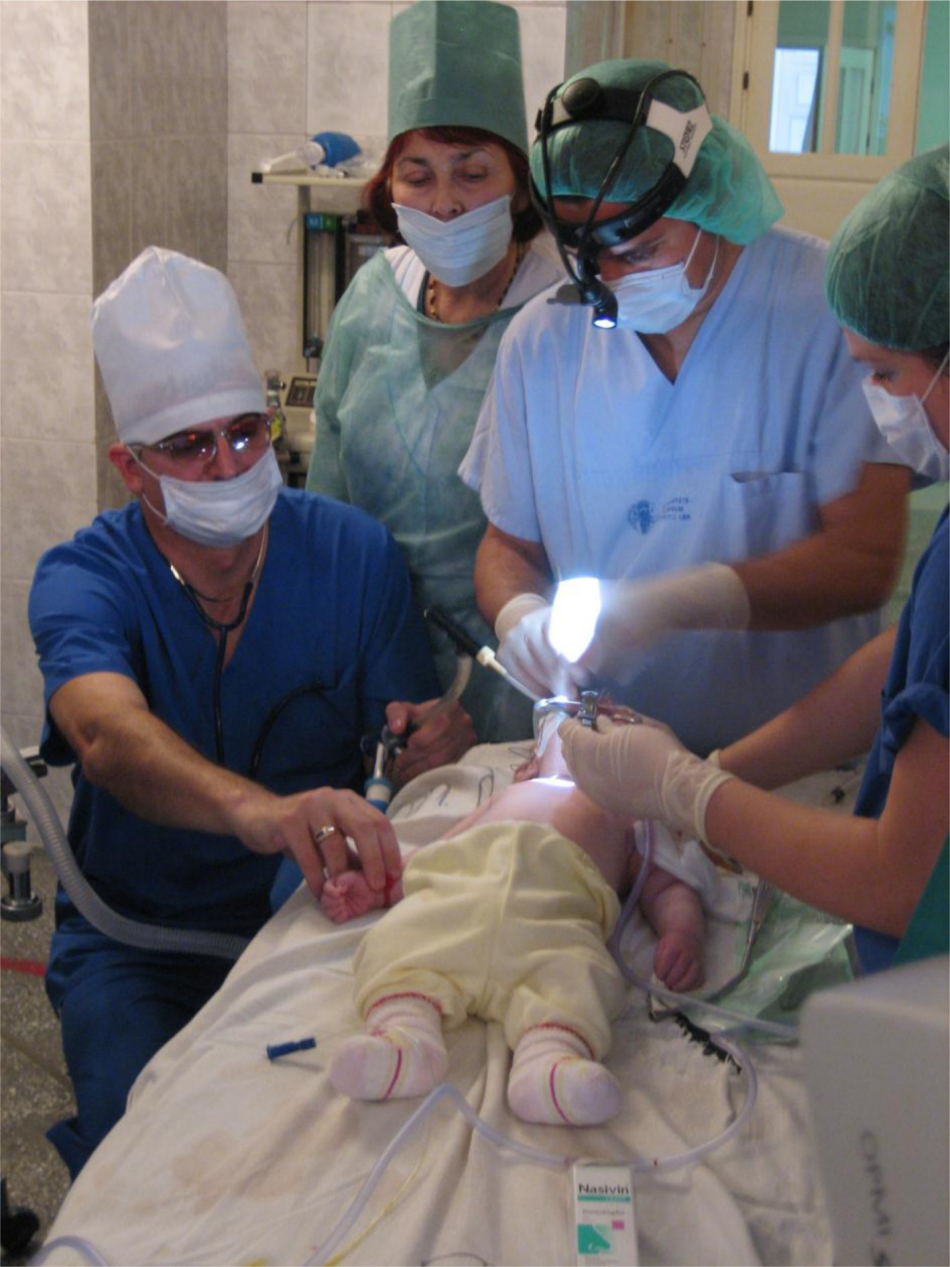
Operating room (OR) arrangement during initial assessment of an infant patient. An international surgeon supervises and trains local surgeons during operations in the OR. Anesthesia and nursing services are provided by local staff. A pulse oximeter was eventually added to the OR equipment to improve patient safety.

In teams of local and international surgeons and staff, 15 complex otologic microsurgical operations for complex middle-ear pathologies (including one non-elective emergency procedure for acute mastoiditis in an infant) were then performed. OR management, anesthesia staff and equipment, and nursing staff were provided by the local hospital (Fig. 1). Local and international surgeons operated using and teaching microsurgical techniques in teams of two, with additional surgeons observing through an operation microscope observer ocular. All cases were documented according to best clinical practices, and patients were followed up regularly, about yearly, by local ENT providers. To clarify clinical findings, local ENT surgeons emailed de-identified photographs, taken by cell phone directly or through the microscope ocular, to international surgeons for further assessment and plans.

Before and during operations, operating surgeons in North Ossetia, both adult and pediatric, received seminars and hands-on training in the OR in otologic microsurgery techniques. As local trainees advanced in their skills and competencies, the role of international surgeons transitioned from supervisor to mentor. At the request of the former, the latter also offered seminar and classroom education to residents and senior staff on advanced topics not already covered by existing surgical education curricula.

We conducted follow-up activities in the subsequent years, during which training continued in a similar format both in Germany and during subsequent project activities in the North Caucasus about once every 2 years.

For this assessment, patients were evaluated clinically and data were extracted from their records 5 years after the initial operations. The recurrence of 5-year postoperative tympanic re-perforations was noted as main postoperative outcome measure. In addition to obtaining consent from all 15 patients or their caregivers, in the absence of a regional ethics review board, we obtained permission from the Vladikavkaz Children's Hospital administration and the North Ossetian Health Ministry to audit the patient charts as part of this evaluation.

## Results

Five female and 10 male patients received major surgical interventions by the international surgical teams. The most common indication was posttraumatic tympanic perforation among Beslan victims (eight patients, 53%), who received tympanoplasties. The remaining patients were not victims of terrorism, but patients who presented with advanced pathologies and who had previously not received adequate surgical treatment due to the health system limitations.

No intra- or postoperative complications related to either surgical procedures or to anesthesia occurred among any of the patients treated. Table 1 shows the recurrence of postoperative tympanic perforations 5 years after the initial operations. Three patients, or 21% of patients who had undergone tympanoplasty, presented with re-perforations, two of which were victims of the Beslan tragedy.

**Table 1:** Outcomes of 15 patients who received otologic operations through the global surgery collaboration

Patient	Age (years)	Gender	Procedure	Diagnosis	Postoperative result
1	40	F	Tympanoplasty type 1, reconstruction of ossicular chain L	Posttraumatic tympanic perforation L	No perforation
2	36	F	Re-tympanoplasty type 1, reconstruction of ossicular chain L	Posttraumatic tympanic perforation L	No perforation
3	6	M	Adenectomy, re-tympanoplasty type 1	Posttraumatic tympanic perforation L	Re-perforation
4	19	M	Tympanoplasty type 1	Chronic otitis media mesotympanica	LTF
5	12	M	Tympanoplasty type 1, reconstruction of ossicular chain with prosthesis	Posttraumatic tympanic perforation	Re-perforation
6	15	M	Re-tympanoplasty type 1, reconstruction of ossicular chain	Posttraumatic tympanic perforation	No perforation
7	14	M	Tympanoplasty type 1	Posttraumatic tympanic perforation R, foreign body	No perforation
8	44	F	Tympanoplasty type 1	Posttraumatic tympanic perforation, tympanosclerosis	No perforation
9	10	M	Adenectomy, tympanoplasty type 1	Chronic otitis media mesotympanica	No perforation
10	12	M	Tympanic tube insertion	Exsudative chronic otitis media, tympanosclerosis	No perforation
11	32	F	Tympanoplasty type 1, reconstruction of ossicular chain R	Posttraumatic tympanic perforation R	No perforation
12	12	F	Adenectomy, tympanoplasty type 1	Chronic otitis media mesotympanica	No perforation
13	0.1	M	Mastoidectomy	Acute mastoiditis	LTF
14	16	M	Re-tympanoplasty type 1, reconstruction of ossicular chain with prosthesis	Chronic otitis media, facial nerve cholesteatoma; status postradical OP L	Re-perforation
15	17	M	Mastoidectomy, tympanoplasty, reconstruction of ossicular chain with prosthesis	Bruton's disease (agammaglobulinemia), chronic otitis media	No perforation

LTF, lost to follow-up.

Four surgeons (three senior and one junior) were trained on-site and enabled to perform and teach microsurgery interventions such as myrinogplasties independently. Further surgeons currently continue to be trained at medical centers in Germany.

## Discussion

### Training and creating access to care are integral components of humanitarian surgery

We report the outcomes of an international partnership to train local providers in otologic microsurgery in a postconflict setting. Our results show that specialized, complex microsurgery can safely be implemented in these settings and lead to surgical results comparable to medical globally. The 5-year postoperative tympanic re-perforation rate of 21% observed in our study is comparable to recent evaluations of larger patient cohorts from major centers, which showed 1-year postoperative tympanic re-perforation rate of 12% in India (9) and a 10-year re-perforation rate of 25% in Spain (10).

Surgical trauma from terrorism does not represent the largest burden of disease in this population. However, responding to a local request to build specialized surgery allowed to create a diagonal program, in which interest in a single disease serves as a mechanism to strengthen a health system as a whole (11). For example, while delivering specialized surgical care, we also created the capacity to provide emergency care (in a case of an infant with mastoiditis) through this collaboration.

Global surgery encompasses not only the safe and effective practice of surgery in resource-limited settings but also the development of systems for surgical care (11). This project focused on system building through humanitarian surgery and specialized surgical training in consideration of local educational needs and resource limitations (12). By training providers and creating capacity, we aimed to provide care to patients who previously had no access. Thus, training local ENT providers (both in the North Caucasus and in Germany) and providing access to specialized care (in the North Caucasus) were integral components of this project to educate the next generation of facilitators of global surgery.

### The majority of surgical burden of disease in postconflict settings is not directly violence-related

Injuries from explosions due to terrorist acts are increasing and have to be managed mostly by non-military care providers outside of combat settings (13). While humanitarian actors often provide care for victims affected by acts of terrorism with limited resources, it is likewise important to address similar burden of surgical disease that is not directly related to violence. We initially planned to electively treat Beslan victims. However, we were soon increasingly confronted with patients who were not primarily affected by trauma but were in need of subspecialty care, which as a consequence of violent conflict has not been available in the region. We also operated a patient who presented with a surgical emergency. Given the lack of available data, we are unable to quantify the exact directly or indirectly conflict-related patient load. Even in conflict areas, the vast majority of surgical interventions are not directly related to violence, but to other pathologies, such as obstetrical emergencies, accidental injury, and infections (14).

Beslan victims sustained multidimensional otologic and other blast injuries: primary injury by the indoor pressure wave; secondary penetrating, blunt or burn trauma from debris; and tertiary injury originating when persons were displaced or surrounding building structures collapsed (7). Psychological trauma is a significant consequence of physical violence and referred to as quaternary blast injuries (15). We found that years after the event, victims’ psychosocial trauma and posttraumatic sequelae – mediated and further exacerbated by daily stressors prevalent in a postconflict society with ongoing violence (16) – impaired our access to patients. Whenever possible when planning surgical interventions in postconflict settings, mental health assessments should be the integral part of surgical strategies and, where appropriate and available, be followed by (brief) interventions and referrals to mental health services.

### Equitable patient selection is key for fair patient care

A fair and transparent patient selection procedure is crucial to ensure equitable patient care. We found patient selection a major challenge in the given setting. In various instances, we saw our workload increased when consulting patients who came with the expectation of being entitled to privileges by the system but for whom treatment was not necessary. We therefore partnered with civil society organizations, to identify and access vulnerable populations in need. In Russia, citizen and mothers groups have often a strong social standing and are often strong advocates for public health. Working with these groups helped us with fair patient selection and the alignment of goals and expectations among stakeholders in this project. This also helped facilitate organizational, procedural, and administrative hurdles even in a highly politicized environment such as the North Caucasus.

**Fig. 2:**
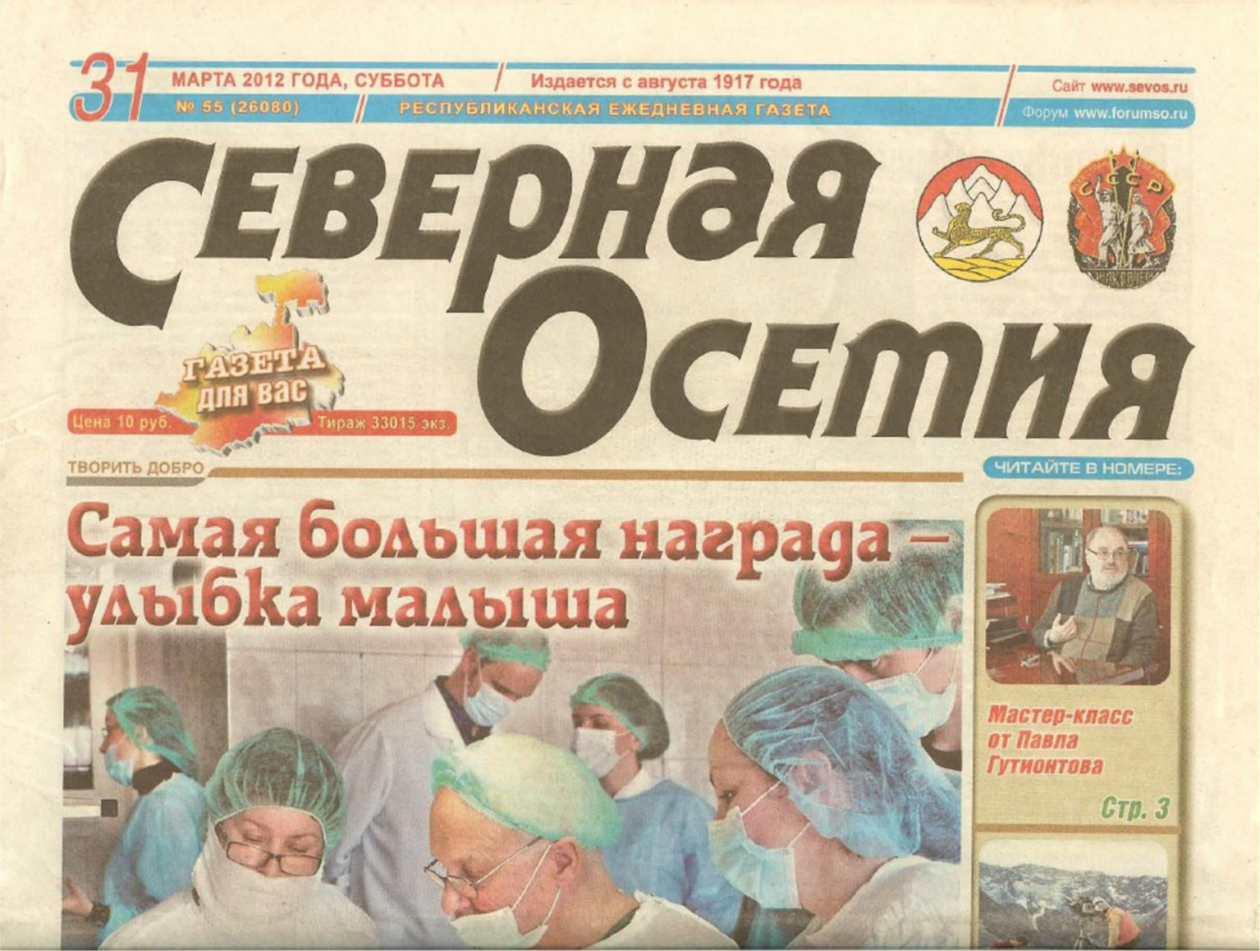
Local media reported on the implementation of surgical services for victims of terrorism. The leading daily newspaper informed potential patients on the title page.

Operating in a health system plagued by corruption and informal payments to obtain care that is officially funded by the state, our goal was to be transparent in our activities. In the course of four follow-up missions since the initial operations reported here, informing the public and political leadership became an important component of our implementation strategy. We maintained systematic communication with the local media, including the leading daily newspaper and the regional television news, to inform the population about our activities and to send messages about seeking surgical assessment (Fig. 2). Local providers assessed care-seeking patients who responded to these messages and referred them to the appropriate follow-up treatment where necessary.

As our results document, we did not experience problems with patient safety or the quality of care provided. We noted that concepts such as evidence-informed practice of medicine and surgery, or quality-improvement approaches are largely foreign concepts in the setting of our collaboration, as they are in most mid-income settings. As we scale-up our activities, an approach to ensure both quality of care as well as fair and adequate patient selection might be the development and use of checklists (17), shown globally to improve surgical outcomes (18). These have been shown to improve quality of care and patient safety in resource-constraint environments (17), and we would argue that fair and appropriate patient selection aided by checklists might be an integral part of surgical care in settings where accountability is problematic.

### Late sequelae from blast injury need to be assessed and addressed long after the event

While the exact local and regional burden of injury and surgical disease is unknown, we assume that otologic operations did not address the largest disease burden. However, in responding to local requests to address otologic sequelae of injuries, we were able to invest in the community and engage in a long-term collaboration with local healthcare professionals – efforts that have been recognized as key to the success of global surgical programs (11). We also learned that following terrorism attacks involving bomb blasts, otologic injury warrants immediate attention.

Although the exact type of explosives used in Beslan remains unknown, we assume that victims held captive in the school gym were exposed to an enhanced blast overpressure. Most of the victims were in close proximity to a wall reflecting the pressure wave, and they all were in a confined, enclosed space at the time of explosion. Besides the gastrointestinal and pulmonary system, the middle and inner ear are at greatest risk of primary blast injury, when the blast pressure wave exerts its main forces at air–tissue interfaces (15).

Ear injury is the most common organ affected after bomb explosions (19). Virtually all individuals situated near (in the case of a terrorist attack in Finland less than 70 m) the center of an explosion suffer some form of ear blast injury (20), particularly in confined spaces as was the case in Beslan, where hostages were held in the school gym. Immediate or at least early treatments within 3 months of the blast trauma yield best results in lesions that do not heal spontaneously (21), whereas complications such as cholesteatoma and ossicular chain discontinuity are common if treatment is delayed (22).

To prevent these sequelae, all victims exposed to explosions, even asymptomatic, should therefore promptly be evaluated with an otoscopic examination for blast trauma to detect tympanic damage (7, 23). However, intact tympanic membranes do not rule out other blast injuries or delayed complications, for example, inner ear problems (7, 24, 25). Since findings can often be subtle, victims should be referred to subspecialists if available. Early treatment of larger lesions is crucial, as is long-term follow-up to detect late complications (26, 27).

### Limitations

Two patients were lost to follow-up, and data are not complete on all patients. The follow-up process of patients complied with local standards, that is, patients were routinely followed-up as they presented to the hospital for outpatient care. In spite of the limited number of patients included in our assessment, it was not feasible to locate all patients to conduct postoperative examinations and audiologic testing after 5 years, partly because the local population includes refugees who moved out of the area.

Given the inherent time and financial constraints of global surgery collaborations, we were not able to collect rigorous costing data to assess the costs of our activities. Total project spending per patient treated (for the cost of trainings and purchase of additional required surgical equipment and consumables) was less than US$ 1,500 per patient, which neither does include travel costs of opportunity costs of using OR time and other local resources nor does it factor in value created through trainings and equipment made available. More comprehensive and robust data are needed to not only demonstrate the feasibility of global surgery in mid-income countries but also its effectiveness and cost-effectiveness (28).

We did not formally assess knowledge and skills of those trained through this collaboration. We plan to design a systematic, longitudinal evaluation of future trainings as we scale up this project.

## Conclusions

In a postconflict, mid-income setting, global surgery collaborations can create local capacity in a safe manner with outcomes that are consistent with global standards. Key element to further capacity building and to reaching the final goal of making microsurgery routinely available as part of the established health services in the region will be an ongoing mutual commitment under local leadership, which can continue to involve international experts.

Partnerships with local health professionals and with civil society organizations are essential for planning and coordination, but also to gain the credibility to approach vulnerable populations who cannot sufficiently be treated within the existing healthcare system. Where resources and accountability are limited, transparent and fair patient selection processes are crucial to address equity aspects of patient care. Future research could explore the use of checklists in humanitarian surgery to facilitate fair patient selection procedures and safe surgery.
